# Optimizing multiplexed imaging experimental design through tissue spatial segregation estimation

**DOI:** 10.1038/s41592-022-01692-z

**Published:** 2022-12-30

**Authors:** Pierre Bost, Daniel Schulz, Stefanie Engler, Clive Wasserfall, Bernd Bodenmiller

**Affiliations:** 1 University of Zurich, Department of Quantitative Biomedicine, Zurich, Switzerland; 2 ETH Zurich, Institute for Molecular Health Sciences, Zurich, Switzerland; 3 Department of Pathology, Immunology, and Laboratory Medicine, Diabetes Institute, University of Florida, Gainesville, FL USA

**Keywords:** Statistical methods, Optical imaging, Software

## Abstract

Recent advances in multiplexed imaging methods allow simultaneous detection of dozens of proteins and hundreds of RNAs, enabling deep spatial characterization of both healthy and diseased tissues. Parameters for the design of optimal multiplex imaging studies, especially those estimating how much area has to be imaged to capture all cell phenotype clusters, are lacking. Here, using a spatial transcriptomic atlas of healthy and tumor human tissues, we developed a statistical framework that determines the number and area of fields of view necessary to accurately identify all cell phenotypes that are part of a tissue. Using this strategy on imaging mass cytometry data, we identified a measurement of tissue spatial segregation that enables optimal experimental design. This strategy will enable an improved design of multiplexed imaging studies.

## Abstract (web summary)

A statistical approach for optimal design of multiplexed imaging studies has been developed. It determines experimental parameters that facilitate cell phenotype identification.

## Main

In the last decade, single-cell technologies for proteomic^1^, transcriptomic and genomic^2–4^ analyses have been developed. Experiments using these technologies have enhanced our understanding of biological systems ranging from human immune cells^5^ to whole cnidarian organisms^6^. Clear guidelines exist to determine the optimal experimental design of sequencing studies, including the total number of cells and sequencing depth necessary for detection of rare cell types or transcripts^7^.

Increasingly, single-cell transcriptomic and proteomic measurements are performed with spatial resolution^8^. Multiplexed imaging techniques are modern counterparts of histological analyses and aim to detect a given set of cell types and their state based on target markers. Therefore, the ability of a multiplexed imaging experiment to recover every expected cell type that is present in a given tissue section is essential. Guidelines for optimal design of multiplexed imaging experiments, such as those performed using imaging mass cytometry (IMC)^9^, MIBI^10^ and CODEX^11^, and in situ hybridization methods, such as sequential fluorescence in situ hybridization (seqFISH)^12^ and multiplexed error-robust fluorescence in situ hybridization (MERFISH)^13^, have not been developed. Given that current highly multiplexed tissue imaging methods have low spatial throughput and high costs, such guidelines, especially to estimate the area to be measured to capture the phenotypic heterogeneity of a tissue, are urgently needed. As it is possible to model the probability of detecting an object when imaging a given area^14^, a solid theoretical foundation for modeling and interpreting the outputs of multiplexed imaging experiments exists. Building on pioneering work on the number of regions that must be imaged to characterize the intensity distribution of a single fluorescent marker and single cell type^15^, we report the development of a strategy to determine the minimal number of fields of view (FoVs) necessary to identify all main cell phenotypes across various healthy and tumor tissues.

## Using spatial transcriptomic data to infer optimal tissue sampling strategy

**Fig. 1:**
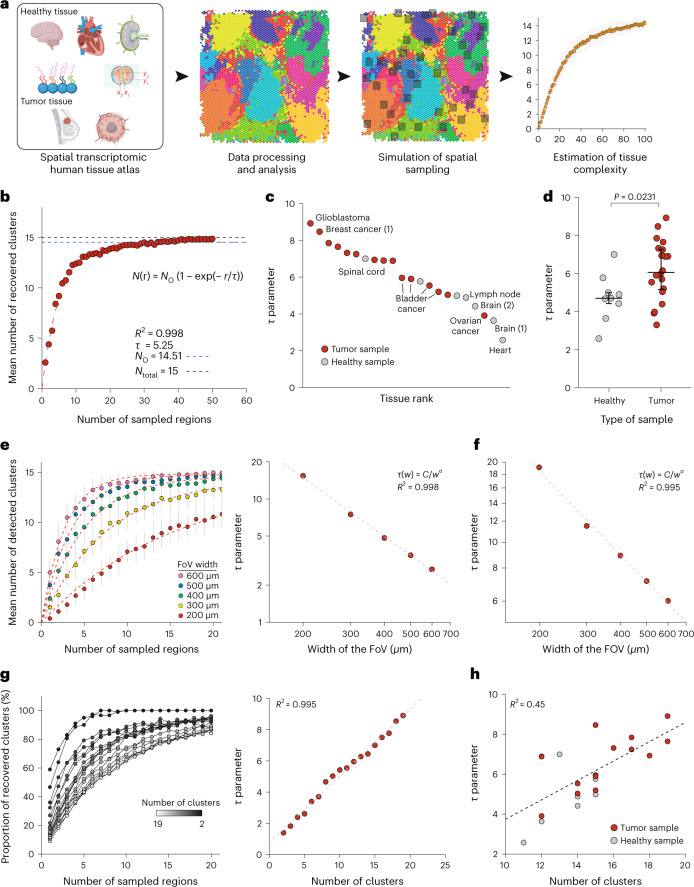
Use of spatial transcriptomic data to determine the optimal tissue sampling strategy for multiplexed imaging. **a**, Analytical workflow used to simulate IMC of human tissues using spatial transcriptomic data. **b**, Number of recovered clusters versus number of sampled regions for a bladder cancer Visium dataset with 400-µm FoVs. Each point corresponds to the mean number of recovered clusters across 50 similar simulations and vertical bars correspond to standard error. The red dashed line corresponds to the fitted function. The horizontal dashed lines correspond to the total number of observed clusters (*N*_o_; blue) and the actual number of clusters (*N*_total_; gray). **c**, Plot of τ for indicated samples from healthy and tumor samples. Numbers in brackets correspond to the number of sample when multiple samples are available for a tissue. **d**, Comparison of τ values from healthy (*n* = 7) and tumor samples (*n* = 15). The *P* value was computed using a two-sided Mann–Whitney rank test. Large bars correspond to the median and small bars to the interquartile range (IQR). **e**, Left, mean number of clusters recovered versus number of sampled regions for FoV widths ranging from 200 to 600 µm for the cerebellum Visium sample. Each point corresponds to the mean number of recovered clusters across 50 similar simulations and vertical bars correspond to the standard error. Red dashed lines correspond to individual fits for each *w* value. Right, relationship between τ and *w* for cerebellum sample. The dashed line corresponds to the linear regression after log_10_ transform. **f**, Relationship between τ and *w* for the glioblastoma Visium sample. The dashed line corresponds to the linear regression after log_10_ transform. **g**, Left, proportion of clusters recovered as a function of τ for a glioblastoma sample for indicated number of clusters. Each point corresponds to the mean number of recovered clusters across 50 similar simulations. For the sake of clarity, the error bars and fitted curves are not displayed. Right, relationship between τ and the number of clusters for a glioblastoma sample. The dashed line corresponds to a linear regression. **h**, Relationship between τ and the number of clusters for all studied samples. The dashed line corresponds to a linear regression. Source data for this figure are provided. Source data

Despite the lack of single-cell resolution, spatial transcriptomic datasets cover large areas of tissues (42 mm2 for Visium arrays^16^) and are assumed to provide an exhaustive description of cell phenotypes present in the tissue. Using these spatial transcriptomic data, we wanted to assess how many FoVs must be measured with another spatial imaging technology to capture all present phenotypes. We used 22 previously collected Visium datasets on 12 different types of tissue (Supplementary Table 1). The Visium data were normalized and clustered to identify different cell phenotypes and cellular niches (Fig. 1a). We then simulated IMC data acquisitions on these same tissues by performing repeated random sampling without replacement of a variable number of non-overlapping, small, square regions with widths of 400 µm (FoV) across the tissue. We computed the number of different clusters (which correspond to unique cell phenotypes) recovered across the sampled regions and aggregated the results across samplings. There was an apparent saturation in the recovery of clusters as the number of FoVs increased (Fig. 1b).

**Extended Data Fig. 1:**
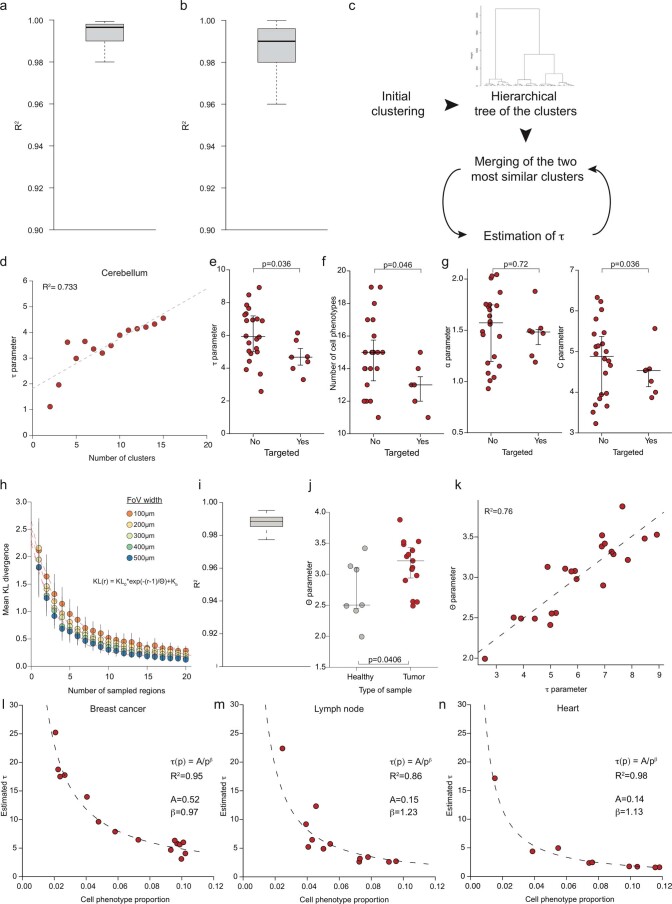
Validation of the statistical model robustness. **(a)** Distribution of R^2^ values for the saturation model described in equation (1) across the Visium datasets. The thick line corresponds to the median, and the bottom and upper limits of the box correspond to the first and third quartiles, respectively. The lower and upper whiskers correspond to the lowest and highest values, respectively, within the range of the first (third) quartile minus (plus) 1.5 times the interquartile range. **(b)** Distribution of R^2^ values for the power-law model described in Equation (2) across the Visium datasets. The lower and upper whiskers correspond to the lowest and highest values, respectively, within the range of the first (third) quartile minus (plus) 1.5 times the interquartile range. **(c)** Approach used to estimate the impact of clustering granularity on τ. **(d)** Relationship between τ and the number of clusters for the cerebellum sample. **(e)** Comparison of the τ parameter value between datasets generated using targeted (n = 7) and un-targeted (n = 22) Visium platforms. The p-value was computed using a two-sided Mann-Whitney rank test. Large bars correspond to the median and small bars to the IQR. **(f)** Comparison of number of cell phenotype detected in datasets generated using targeted (n = 7) and un-targeted (n = 22) Visium platforms. The p-value was computed using a two-sided Mann-Whitney rank test. Large bars correspond to the median and small bars to the IQR. **(g)** Comparison of the alpha (left panel) and **C** (right) parameters between datasets generated using targeted (n = 7) and un-targeted (n = 22) Visium platforms. The p-value was computed using a two-sided Mann-Whitney rank test. Large bars correspond to the median and small bars to the IQR. **(h)** Mean KL divergence between sampled and total cell composition vs number of sampled regions for FoVs ranging from 100 to 500 µm for a Visium breast cancer dataset. Each point corresponds to the mean number of recovered cluster across 50 similar simulations, and vertical bars correspond to the standard error. The red dashed lines correspond to individual fits for each *w* value. **(i)** Distribution of R^2^ values for the KL sampling model described in Equation (3) across the Visium datasets. The lower and upper whiskers correspond to the lowest and highest values, respectively, within the range of the first (third) quartile minus (plus) 1.5 times the interquartile range. **(j)** Comparison of θ values from healthy (n = 7) and tumor samples (n = 15). The p-value was computed using a two-sided Mann-Whitney rank test. Large bars correspond to the median and small bars to the IQR. **(k)** Comparison of θ and τ parameter values across the Visium samples. **(l), (m) and (n)** Relation between individual phenotypes τ and their abundance in three Visium datasets, breast cancer, lymph node and heart respectively. Dashed black line corresponds to the power-law model described in Equation (4). Each dot corresponds to a specific cell phenotype. Source data

To model the relationship between the number of clusters and the number of FoVs, we used a model derived from the analysis of homogeneous Poisson point processes:^14^

1 $$N\left( {\mathrm{r} }\right) = N_{\mathrm{O}}\left( {1 - \exp \left( { - \frac{r}{\tau }} \right)} \right)$$

where *r* is the number of FoVs, *N*(r) is the mean number of recovered clusters, *N*_O_ corresponds to the total number of observed clusters and τ indicates how many regions must be imaged to recover most of the known cell phenotypes. According to this model, 2τ FoVs must be imaged to recover 86% of known clusters. This model fitted well across all Visium datasets (Extended Data Fig. 1a) and τ varied significantly across tissues (Fig. 1c). We observed that tumor samples had higher τ values than healthy samples, indicating that more FoVs are required on average to identify cell phenotype clusters in tumor tissue than in healthy tissue (Fig. 1d, *P* = 0.0075).

We next studied the effects of the width of the FoV, *w*, on spatial sampling efficiency by performing the same simulated IMC analysis with various values of *w*. As expected, fewer regions needed to be imaged to recover all known clusters when *w* values were larger (Fig. 1e, left panel). Following logarithmic transformation, there was a linear relationship between *w* and τ across all studied tissues (Fig. 1e, right panel, Fig. 1f and Extended Data Fig. 1b), indicating an underlying power law. Therefore, τ can be written as a function of *w*:

2 $$\tau \left( w \right) = \frac{{{C}}}{{{{w}}^{\upalpha}}}$$

where *C* and α are two positive constants and depend on the sample.

We then explored whether there was a relationship between τ and the granularity of the initial clustering analysis. To test this, we aggregated the most similar pairs of cell phenotypes for each sample by determining the correlation between mean expression profiles, performed a sampling analysis to compute τ and then merged the next two most similar clusters, repeating until only two clusters remained (Extended Data Fig. 1c). We observed a linear relationship between τ and the number of clusters in certain Visium samples, such as a glioblastoma (Extended Data Fig. 1g), but the linear regression fit poorly for others, such as cerebellum (Extended Data Fig. 1d), indicating that this relationship could not be generalized. In addition, across all samples, τ and the total number of observed cell clusters were moderately correlated (Fig. 1h, *R*^2^ = 0.45), implying that the number of clusters notably impacts the value of τ and explains the difference in τ values between healthy and tumor samples.

In addition, we investigated whether changing the number of features measured in a spatial experiment affects the τ parameter. To do so, we performed a sampling analysis on seven human datasets generated using a targeted version of the Visium platform where only 1,000 genes are measured. Interestingly, we observed that the estimated τ values were significantly lower in the targeted samples compared to the non-targeted samples (Extended Data Fig. 1e). We hypothesized that this might be due to the reduced diversity of cell phenotypes measured by the targeted Visium platform; indeed, we observed a significantly reduced number of cell phenotypes recovered in targeted samples compared to untargeted samples (Extended Data Fig. 1f). Moreover, no significant difference of the α parameter value was observed between the two types of samples (Extended Data Fig. 1g, left panel), whereas the *C* parameter was lower in the targeted samples. Thus, changing the features measured can significantly alter the τ parameter by altering the number of detected cell phenotypes.

To further assess our approach, we measured the Kullback–Leibler (KL) divergence between the estimated proportions of cell phenotypes from a given number of sampled FoVs and the real proportions computed using the whole dataset. We observed that the KL divergence substantially decreases when increasing the number of sampled regions (Extended Data Fig. 1h). We fit an equation similar to equation (1), where the KL divergence is a function the number of sampled FoVs (Methods), to all samples and observed a persistent high quality of the model with an *R*^2^ above 0.97 for all samples (Extended Data Fig. 1i), as well as a higher value of the θ parameter, the equivalent of the τ parameter, in tumor samples compared to healthy samples (Extended Data Fig. 1j, *P* = 0.046). We observed a strong correlation (*R*^2^ = 0.76) between the matched θ and τ parameter values (Extended Data Fig. 1k). These results further support the value of our initial approach that models the number of regions to be sampled based on cell phenotype recovery.

Lastly, we investigated whether it was possible to infer the τ value of a given tissue using only the composition of the tissue, as would be available from single-cell RNA sequencing data. To do so, we computed the τ value of each individual cell phenotype for three different tissues (breast cancer, lymph node and heart; Methods) and studied the relationship of these values with the respective cell phenotype abundance within each tissue. We observed that a simple power-law model was able to efficiently link cell phenotype abundance and individual τ values (Extended Data Fig. 1i–k;Methods); however, the parameters controlling this relationship varied across the three tissues, suggesting that cell composition alone is insufficient to predict the result of a given sampling in a tissue and that spatial data are needed. Indeed, predicting the individual τ values for each cell phenotype from breast cancer using the model computed from cardiac tissue data resulted in a two-fold underestimation compared to observed values in breast cancer (mean ratio of 0.44).

## Characterization of α and C as measures of tissue spatial segregation

**Fig. 2:**
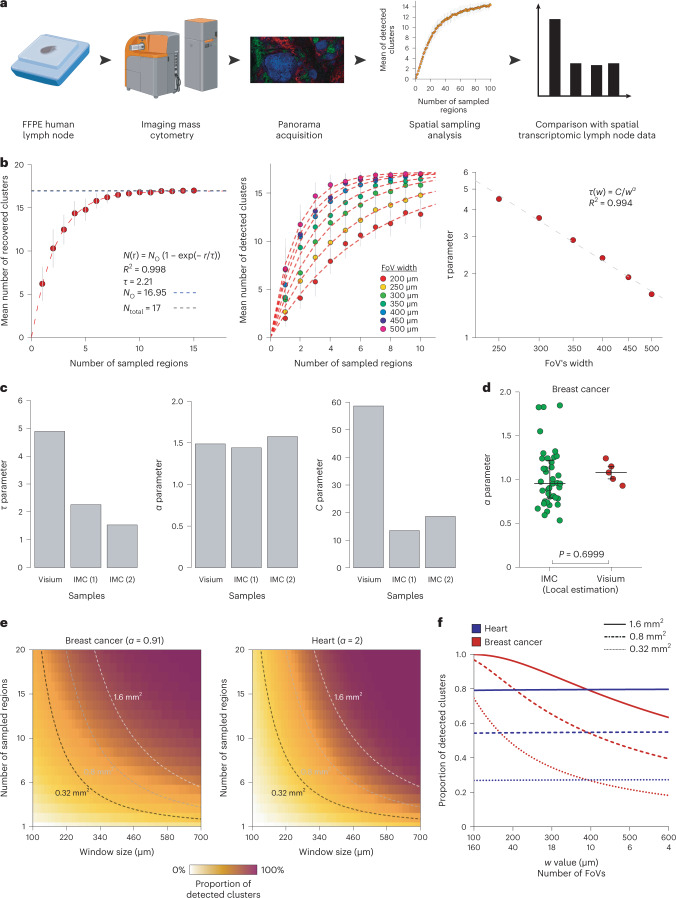
Identification of a technology invariant measure of tissue complexity. **a**, Experimental workflow to compare the results of spatial transcriptomic and IMC large-scale analysis. **b**, Left, number of recovered clusters versus number of sampled regions for IMC lymph node data from sample no. 1. Each point corresponds to the mean number of recovered clusters across 50 similar simulations and vertical bars correspond to the standard error. The red dashed line corresponds to the fitted function. The horizontal dashed lines correspond to the number of observed clusters (*N*_o_; blue) and the actual number of clusters (*N*_total_; gray). Middle, number of recovered clusters versus number of sampled regions for FoVs ranging from 200 to 500 µm for the IMC lymph node data from sample no. 1. Each point corresponds to the mean number of recovered clusters across 50 similar simulations and vertical bars correspond to the standard error. The red dashed lines correspond to individual fits for each *w* value. Right, relationship between τ and *w* for the IMC lymph node data from sample no. 1. The dashed line corresponds to the linear regression after log_10_ transform. **c**, Left, values of τ for 400-µm width FoV for the Visium and IMC datasets for lymph node samples. Middle, values of α for the lymph node datasets. Right, values of C for the lymph node datasets. **d**, Comparison of α values between the IMC breast cancer dataset (*n* = 39 FoVs) and the five Visium breast cancer datasets (*n* = 5 samples). The *P* value was computed using a two-sided Mann–Whitney rank test. Large bars correspond to the median and small bars to the IQR. **e**, Estimation of sampling strategy efficiency for breast cancer (left) and heart (right) Visium samples. The dashed lines correspond to the possible values taken for a fixed area surface. **f**, Proportions of recovered clusters when the area imaged (indicated by solid, dashed or dotted lines) was fragmented for breast cancer (red lines) and heart (blue lines) datasets. Source data for this figure are provided. Source data

**Extended Data Fig. 2:**
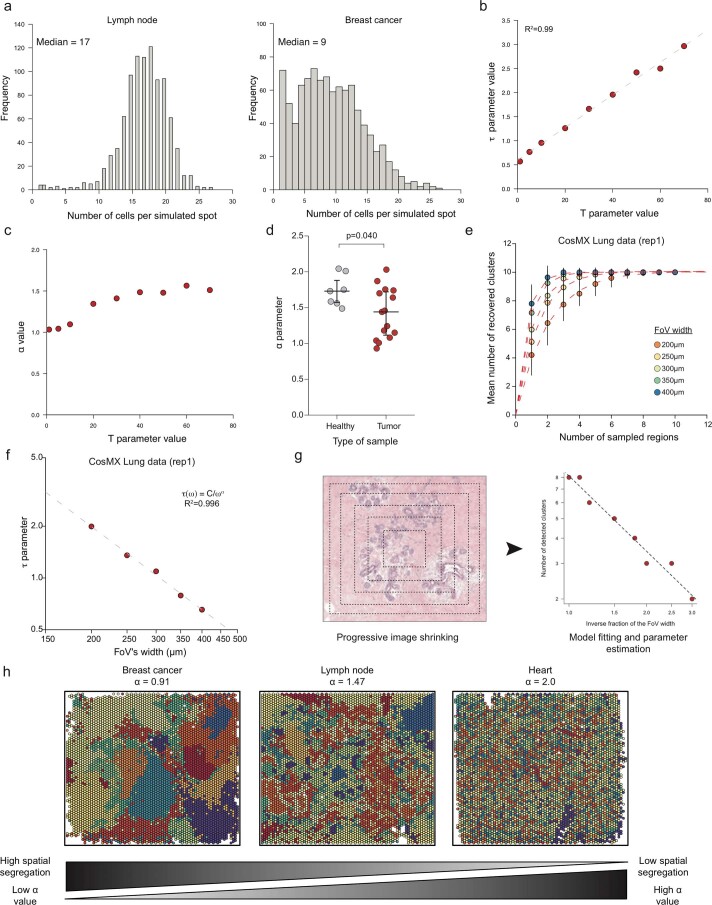
Spatial segregation analysis across platforms. (**a**) Distribution of the number of individual cells in regions of the size of Visium capture spots in a lymph node (left) and a breast cancer sample (right). (**b**) Relationship between the threshold T parameter and the τ value for a FoV’s width of 400 µm in the IMC lymph node sample (1). The dashed line corresponds to an ordinary least square regression. (**c**) Relationship between the threshold T parameter and the α value for the IMC lymph node sample (1). (**d**) Plot of α values from healthy (n = 7) and tumor (n = 15) samples. The p-value was computed using a two-sided Mann-Whitney rank test. Large bars correspond to the median and small bars to the IQR. (**e**) Sampling analysis of the first CosMX lung sample replicate. Each dot corresponds to the mean number of clusters recovered vs number of sampled regions for FoV widths ranging from 200 to 400 µm and vertical bars correspond to the standard error. (**f**) Relationship between τ and *w* for the CosMX lung dataset. The dashed line corresponds to the linear regression after log10 transform. (**g**) Approach used to estimate α from a set of small IMC FoVs. (**h**) Relationship of cell phenotype spatial segregation with α. Each panel represents a different Visium sample where each point corresponds to a capture spot and is colored based on its associated cell phenotype. Source data

We went on to assess whether results obtained from the analysis of spatial transcriptomic data can be generalized to data obtained from other technologies. In particular, we focused on technologies with a single-cell resolution. We imaged large areas (4.44 and 6.13 mm^2^) of two human formalin-fixed paraffin-embedded lymph node sections using IMC with two antibody panels (Supplementary Table 2) and performed a spatial sampling analysis with various FoV widths (Fig. 2a). To have comparable analysis between Visium and IMC data, we determined that a single Visium capture spot corresponds to a median of 17 and 9 cells in lymph node and breast cancer IMC data respectively (Extended Data Fig. 2a). Based on this, we adjusted the threshold for cell phenotype recovery, *T*, for each data type to roughly equalize the number of cells that must be recovered (Methods). As for the Visium lymph node data, the relationship between the number of sampled regions and recovered clusters was fit by equation (1) (Fig. 2b, left panel), and the FoV width affected *τ* as described by equation (2) (Fig. 2b, middle and right panels). However, the values of τ differed between the Visium lymph node data and the two IMC datasets (Fig. 2c, left panel), indicating that a different number of regions must be sampled from the two datasets to recover the same fraction of cell phenotypes. When analyzing the parameters of equation (2), we found that the α parameter did not differ between the two imaging modalities (Fig. 2c, middle panel), whereas there was a large difference in C (Fig. 2c, right panel). To check that our analysis was robust to the choice of the recovery parameter T (Methods), we repeated it with various values and observed that while it strongly impacts the inferred τ value (Extended Data Fig. 2b), the computed α parameter is constant for T from 20–70 cells (Extended Data Fig. 2c).

Within a given technology, the parameter α varied considerably across tissue types, with values ranging from 2.00 for cardiac tissue to 0.91 for breast tumor tissue analyzed by spatial transcriptomics (Extended Data Fig. 2d). As for τ at a given FoV width, α was significantly lower in cancer samples than healthy tissues (Extended Data Fig. 2d, *P* = 0.0401). In a separate validation, our model also fit well to a lung tumor dataset generated using highly multiplexed single-molecule fluorescence in situ hybridization (Nanostring CosMX) and the relation between τ and *w* was also conserved (Extended Data Fig. 2e,f).

To further assess the properties of α, we re-analyzed a previously published IMC dataset^17^ containing 100 FoVs, each derived from a unique breast cancer sample. For each FoV, we simulated a progressive shrinkage of the width and computed the effect on the number of detected clusters to obtain an estimate of α for each FoV (Extended Data Fig. 2g, Methods). We did not observe a significant difference between the estimated α values using IMC data and the one estimated from five different Visium breast cancer samples (Fig. 2d, *P* = 0.699). These results support a hypothesis that α is a technology-independent but tissue-dependent parameter. Intuitively, α can be seen as a measure of cell phenotype spatial segregation (Extended Data Fig. 2h): for a high value of α (α = 2), such as in cardiac tissue, cell phenotypes are randomly spatially spread and do not segregate (that is, cluster) together. Conversely, tissues with a low α value display large patches of identical cell phenotypes, making the sampling of large FoVs unproductive.

To evaluate the impact of α on the sampling strategy design, we computed the theoretical number of recovered clusters when sampling a defined area with various numbers and area of FoVs. We performed this analysis on two different types of tissue—cardiac (low spatial segregation, α = 2) and breast cancer tissue (strong spatial segregation, α = 0.91)—using the values fitted on Visium datasets. First, we observed that the number of recovered clusters was not affected by the fragmentation of the FoVs for the heart sample but only by the total imaged area (Fig. 2e,f). In contrast, for breast cancer samples, increasing the fragmentation of the imaged area into multiple small regions of interest increased the number of recovered cell clusters; for instance, when imaging 0.8 mm^2^, shifting from four to ten regions of interest will result in the doubling of the proportion of recovered cell phenotypes, from less than 40% to more than 80% (Fig. 2e,f).

## Discussion

Here we report how experimental design parameters impact the efficiency of multiplexed imaging experiments to recover all present cell phenotypes using the proportion of recovered cell phenotypes as a simple yet robust metric. Our analysis identified the number of FoVs and their widths as key parameters that drive cell phenotype recovery. Moreover, we determined the precise mathematical relationship linking these two parameters to the number of recovered clusters. We found that the impact of FoV width on the experiment efficiency was regulated by a term α that seems to be tissue specific and potentially independent of the imaging technology used. In practice, α can be estimated in a pilot experiment using either a spatial transcriptomic approach or by imaging a large region (several mm^2^) of a representative sample of a given cohort using a multiplexed imaging technology, ideally the same as the one planned to be finally used (Supplementary Information). Once determined for a tissue type, α should be valid for other tissue samples of similar type as long as the recovery threshold is in the same order of magnitude as the one initially used to compute α.

Interestingly, we observed highly variable values of α across tissues and this must be taken into account when planning a multiplexed imaging experiment. Indeed, a value of α close to two means that one can image a small number of large FoVs or many small FoVs and recover the same number of cell phenotypes. In contrast, a small α value requires the sampling of many small regions to efficiently recover the maximum number of cell phenotypes at a minimal cost. To facilitate the planning of imaging experiments, we provide α values for various healthy and cancerous tissues (Supplementary Table 3).

Our model has limitations. Currently, it only determines the ideal parameters to capture all, or almost all, phenotypic clusters present in the tissue. The model does not consider spatial relationships and tissue structures such as tertiary lymphoid structures or pairs of interacting cells. Indeed, preliminary analysis revealed that our model was unable to generally describe the sampling of cell–cell interactions within a lymphoid tissue. Additional work is therefore needed to see how our results, which are cell-based, can be extended to multi-cellular structures to implement more complex multiplex imaging experiments. In addition, while robust to minor parameter values changes, the results of our analyses could be impacted by changes to the cell phenotype recovery threshold (that is, the number of cells of a given phenotype that must be recovered for the phenotype to be considered as recovered). Specifically, definition of α as invariant for a given tissue is robust within a recovery threshold of 20–70 cells; outside of this range, α estimation might be unreliable, and values outside of this range should therefore not be used with this model. Also, the recovery threshold will affect *τ* and should, therefore, be taken into account when designing sampling strategies in a new experiment. Lastly, the model assumes that cells are present in tissue at similar densities, a hypothesis that seems true for the samples we analyzed as most cell phenotypes represent between 4 and 12% of total cell composition (Supplementary Fig. 1i–k). However, in the case of samples where cell phenotype proportions span across several orders of magnitude, the model is unable to hold, resulting in ill-fitting and unreliable results.

Beyond application to the design of multiplexed imaging experiments, our results could also be used in the field of anatomical pathology, wherein the current standard for the classification of samples is the analysis of one to four circular punches of variable diameter (600 µm to 2 mm)^18^. Although we focused on the recovery of multiple cell phenotypes rather than a single type of cell (for instance, HER2^+^ cells in breast cancer samples)^19^, it is likely that a similar phenomenon of spatial segregation determines the efficacy of this type of sampling. In summary, our approach provides essential guidance for the study of tissue structures using multiplex imaging in a time and cost-efficient manner.

## Methods

Lymph nodes were obtained from organ donors under research consent from University of Florida Institutional Review Board (IRB201600029). These were obtained as for transplant with central flushing after cross-clamp before excion from the mesentery. The tissue was then shipped on wet ice in UW buffer (University of Wisconsin transplant buffer) to the university of Florida before being fixed in 10% Neutral Buffered Formalin for 16 hours before embedding paraffin.

### Visium data preprocessing and analysis

Visium data were downloaded from the 10x Genomics website (support.10xgenomics.com/spatial-gene-expression/datasets/), the Gene Expression Omnibus (GEO) or the Zenodo data portals. Individual access numbers are provided in Supplementary Table 1. Spots with less than 1,000 unique molecular identifiers and genes with less than 100 unique molecular identifiers were removed before any analysis. Data were analyzed by combining the classical single-cell RNA sequencing pipeline Pagoda2 (ref. 20) with a latent Dirichlet allocation analysis step. Briefly, the top 1,500 most variable genes were identified using the ‘adjustvariance()’ function from the Pagoda2 package and the raw count data matrix containing only these genes was processed using the ‘FitGoM()’ function from the CountClust package, with a tolerance parameter set to 100 and the number of topics set to 5, 10, 15 or 20. For each number of topics, the Bayesian Information Criterion score was computed and the number of topics displaying the lowest Bayesian Information Criterion or an elbow-like inflection was selected. The mixing matrix was then used for the next steps of analysis. A *k*-nearest-neighbor graph was built using the ‘makeKnnGraph()’ function, with parameter *k* set to 15 and using a cosine distance before performing a community detection analysis with the ‘getKnnClusters()’ function with default parameters (corresponding to Louvain’s community detection^21^).

### Analysis of targeted Visium datasets

Targeted Visium datasets were downloaded from the 10x Genomics website (support.10xgenomics.com/spatial-gene-expression/datasets/). Detailed of the downloaded samples are available in Supplementary Table 1.They were analyzed using the same computational strategy as for the regular Visium samples, including an LDA-based dimensionality reduction step, except that all genes were used and no variance-based gene selection was performed.

### IMC data preprocessing and analysis

The raw .MCD (MathCad document) files were processed using the Steinbock pipeline, v.0.70^22^. In brief, the raw files were converted into .tiff files, and the cells were segmented using a pretrained neural network^23^ using the H3K9ac channel as the nuclear channel and CD45RA/RO and Vimentin as the membrane/cytoplasmic channels. Default parameters were used for Mesmer with the exception of the --type parameter, which was set to ‘nuclei’. The mean channel intensity was then computed for each cell and exported as a text file, together with the location, the size and other basic information on the cells. The single-cell IMC data were then analyzed using in-house R scripts (https://github.com/BodenmillerGroup/MI_Sampling_study/blob/main/List_scripts_IMC_processing, R version 4.0.3). Each channel was normalized by performing a Poisson regression between the total channel intensity and the cell size (in pixels); the Pearson’s residuals were extracted as the new scaled values. The cells were then clustered by first building a *k*-nearest-neighbor graph with 15 neighbors (using cosine distance) and then clustered using Louvain’s community detection implemented in the igraph package with default parameters^24^.

### Processing and analysis of CosMX data

Data corresponding to the three lung cancer CoxMX samples were downloaded from https://nanostring.com/products/cosmx-spatial-molecular-imager/ffpe-dataset/ (three replicates of Lung5 sample). Data were processed using the same approach as for IMC data except that only the 200 most variable genes were selected by computing the multinomial deviance *D* of each gene, the deviance of a given gene expression vector *x* over *m* cells being defined as:

$$\begin{array}{*{20}{l}} {D\left( x \right)} \hfill & = \hfill & {\mathop {\sum}\limits_{i = 1}^m {x_i{\mathrm{log}}\left( {x_i} \right) + n{\mathrm{log}}(n)} } \hfill \\ {} \hfill & {} \hfill & {{\mathrm{with}}\,n = \mathop {\sum}\limits_{i = 1}^m {x_i} } \hfill \end{array}$$

*x*_*i*_ being the number of RNA molecules in cell *i*and *n* the total number of RNA molecules detected for this gene. The 200 genes with the highest deviance were selected and used for further analysis. Following the processing of the data, a regular sampling analysis was performed on each sample as described above.

### Spatial sampling analysis

To simulate spatial sampling strategies, we created a simple function that iteratively selects a random point on the sample, ‘draws’ a square with the sampled point at the center and then checks whether this square overlaps with previously sampled squares. In case of overlap, the point is removed and a new point is sampled. A cluster was considered as detected by a given spatial sampling (set of sampled FoVs) if more than *T* spots belonging to that cluster were located in the drawn squares. The threshold *T* was changed based on the type of data: it was set to three spots for Visium data, 50 cells for the IMC lymph node and CosMX lung cancer data, and 20 cells for the IMC breast cancer data. As an individual Visium spot has a diameter of 50 µm, one can assume that between 10 to 20 cells can be mapped to a single spot, especially in highly dense tissues. Therefore, we chose the threshold of three spots so that the results obtained from Visium data could be compared to the one from IMC data. This sampling was repeated 50 times to obtain a robust estimate.

The model proposed in equation (1) was derived from the analysis of a homogenous Poisson point (HPP) process defined by a density parameter *ƛ*. The probability that a random square of size *w* contains no points is equal to exp(−*ƛw*^2^). A basic property of HPP processes is that the probability of finding no points in *r* independent (that is, non-overlapping) squares is exp(−*ƛrw*^*2*^), and therefore the probability of finding at least one point is 1 − exp(−*ƛrw*^*2*^). As we examined *N*_O_ different cell phenotypes (that is, *N*_O_ different and independent point processes), the mean number of recovered phenotypes for a fixed number of squares *N*(r) is *N*_O_ × (1 − exp(−*ƛrw*^*2*^)), thus justifying the use of equation (1). While in practice each cell phenotype has a different *ƛ*, that is, density and value, it is important to note that many cell phenotypes have an abundance between 4% and 12% (Extended Data Fig. 2i-k) and that they share similar individual τ values. Therefore, while our model does not reflect the true complexity of the data, it is a valid and easy-to-use approximation that provides key information on the best sampling strategy. Lastly, *N*_O_ corresponds to the maximal number of cell phenotypes that can be recovered—that is, the maximum number of cell phenotypes in excess to the used recovery threshold.

To fit the model described in equation (1), we used the ‘nls()’ function. The exact R command initially used is:

$$nls\left( {y\sim N \ast \left( {1 - {\mathrm{exp}}\left( { - x/tau} \right)} \right),\,{\mathrm{start}} = {\mathrm{list}}\left( {N = 20,\,tau = 5} \right)} \right)$$

where *x* represents the number of FoVs and *y* represents the mean number of clusters recovered. The *tau* parameter corresponds to the τ parameter described in equation (1) and *N* corresponds to the *N*_O_ parameter also described in equation (1). The optimization is done using the default Gauss–Newton algorithm. The quality of the fit was estimated using ‘cor()’ and ‘predict()’ functions.

Fitting equation (2) to the data was done by first applying a log_10_ transform to the data and then performing a classical least square regression using the ‘lm()’ R function. The R code used is:

$$lm\left( {{\mathrm{log}}10\left( {tau} \right)\sim{\mathrm{log}}10\left( w \right)} \right)$$

where *tau* corresponds to a numerical vector containing the τ values for different FoV widths and *w* to the corresponding FoV width values.

To study the effects of clustering granularity on cluster recovery, we first computed the mean expression of each gene in each cluster, then built a hierarchical clustering tree using Euclidean distance and Ward’s criterion. Then, using this tree, we iteratively merged the different clusters. At each step, we performed a spatial sampling analysis.

### Estimating the number of cells within a single Visium capture spot

To estimate the number of cells present in a single Visium spot we performed random sampling of small square FoVs with the same area as a Visium spot, that is, 44-µm wide squares. We sampled 1,000 FoVs on the IMC lymph node sample (1) and ten FoVs for each individual FoV of the IMC breast cancer dataset, resulting in 1,000 total FoVs.

### Modeling cell phenotype proportion recovery

Similarly to the spatial sampling analysis described above, we randomly sampled a given number of non-overlapping FoV and quantified the number of spots belonging to each cell phenotype. For each independent sampling, we transformed the obtained count vector to a proportion vector by dividing by the total number of sampled cells and computed the corresponding KL divergence

$${\mathrm{KL}}\left( {P||Q} \right) = {\sum} {P_i\,{{{\mathrm{log}}}}\left( {\frac{{P_i}}{{Q_i}}} \right)}$$

where *P* is the sampled proportion vector and *Q* is the proportion vector computed using the full dataset.

To link the KL divergence and the number of sampled FoV, we used the following equation:

3 $${\mathrm{KL}}\left( r \right) = {\mathrm{KL}}_{\mathrm{O}}\exp \left( { - \frac{{r - 1}}{\theta }} \right) + {\mathrm{KL}}_{\mathrm{b}}$$

Where *r* corresponds to the number of FoVs sampled, KL(*r*) to the mean KL divergence when *r* FoVs are sampled, and KL_O_, KL_b_ and θ are parameters to determine. KL_O_ can be interpreted as the mean KL divergence observed with one FoV, and θ corresponds to how many regions must be imaged to have a low KL divergence. Lastly, KL_b_ is the baseline KL divergence, that is, the minimal KL divergence observed.

To fit the model described in equation (3), we used the ‘nls()’ function. The exact R command initially used is:

$$\begin{array}{l}{\mathrm{nls}}\left( \right.{y\sim{\mathrm{KL}}\_{\mathrm{O}}\ast {\mathrm{exp}}\left( { - \left( {x - 1} \right)/theta} \right) + {\mathrm{KL}}\_{\mathrm{b}},\,{\mathrm{start}}} \\= {\mathrm{list}}\left( {theta = 2,{\mathrm{KL}}\_{\mathrm{O}} = 1,{\mathrm{KL}}\_{\mathrm{b}} = 0.01} \right)\left. \right)\end{array}$$

where *x* represents the number of FoVs and *y* the mean KL detected. The *theta* parameter corresponds to the θ parameter described in equation (3) while KL_0 and KL_b correspond to the KL_O_ and KL_b_ parameters respectively, also described in equation (3). The optimization is done using the default Gauss–Newton algorithm. The quality of the fit was estimated using ‘cor()’ and ‘predict()’ functions.

### Predicting τ using cell phenotype abundance

For each individual dataset, we computed the abundance of each phenotype by dividing the number of spots belonging to each phenotype by the total number of phenotypes in the dataset. We then performed a sampling analysis as described above, but then studied the probability of recovering each cell phenotype individually. To do so, we fitted a modified version of equation (1) where *N*_O_ was set to one, using the ‘nls()’ function with τ starting value being equal to two.

In the case of HPP, the relation between τ and λ is equal to:^14^

$$\tau \left( \lambda \right) = A/\lambda$$

where A is a positive constant.

We decided to use a slightly more flexible model described in equation (4):

4 $${\tau} \left( p \right) = A/p^{\beta}$$

Where *p* represents the normalized abundance of a given phenotype and *A* and β are two positive constants. Fitting equation (4) to the data was done by first applying a log_10_ transform to the data before performing a classical least square regression using the ‘lm()’ R function. The R code used is:

$$lm\left( {{\mathrm{log}}{10}\left( {tau} \right)\sim{\mathrm{log}}{10}\left( p \right)} \right)$$

where the *tau* variable corresponds to a numerical vector containing the cell phenotype individual τ values and *p* the numerical vector containing the cell phenotype abundance.

### Lymph node section processing and IMC data acquisition

The two lymph node formalin-fixed, paraffin-embedded blocks were first cut into 5-µm thick sections. They were then dewaxed and rehydrated and subjected to a heat-induced epitope retrieval step for 30 min at 95 °C in 10 mM Tris, pH 9.2, 1 mM EDTA. The sections were then incubated in blocking buffer (3% BSA in TBS-T) for 1 h at room temperature, before incubation with antibodies (diluted in blocking buffer) overnight at 4 °C. Nuclear staining was then performed by adding an iridium solution (5 nM) diluted in TBS (1:100 dilution) to the sample and incubating for 5 min. The samples were then washed three times (10 min per wash) in TBS and dried. Images were acquired using an Hyperion Imaging System, with the ablation frequency set to 200 Hz and the ablation energy set to 6 dB, with X and Y steps set to 1 µm.

### Breast cancer IMC data re-analysis

The SingleCellExperiment object containing single-cell information from 100 FoVs, each one derived from a different sample, was downloaded from the Zenodo platform (10.5281/zenodo.3518284) and analyzed using the following strategy: we first aggregated all cancer clusters (clusters 14 to 27) into a single cluster as the cancer clusters displayed a strong patient specificity. For each FoV, we progressively reduced the size of the image by factors of 1.1, 1.2, 1.5, 1.8, 2.2, 2.5 and 3.0 and computed the number of recovered clusters. We then performed a linear regression between the log-transformed number of recovered clusters and the size of the reduced FoV using the ‘lm()’ core function. FoVs with a low-quality model (*R*^2^ < 0.9) were removed and the slope of the regression was taken as the estimate of α. If we combine equations (1) and (2) when considering a single FoV, we have:

$$N\left( 1 \right) = N_{\mathrm{O}}\left( {1 - {{{\mathrm{exp}}}}\left( {\frac{{ - 1}}{\tau }} \right)} \right)$$

Therefore:

$$\log \left( {1 - \frac{{N\left( 1 \right)}}{{N_{\mathrm{O}}}}} \right) = - \frac{1}{\tau } = \frac{{ - w^\alpha }}{C}$$

$${\mathrm{as}}\,N\left( 1 \right) < < N_{\mathrm{O}},\,{\mathrm{we}}\,{\mathrm{have}}\,\log \left( {1 - \frac{{N\left( 1 \right)}}{{N_{\mathrm{O}}}}} \right) \approx - \frac{{N\left( 1 \right)}}{{N_{\mathrm{O}}}}$$

$$\log \left( {N\left( 1 \right)} \right) \approx \alpha \log \left( w \right) + \log \left( {N_{\mathrm{O}}} \right) - {{{\mathrm{log}}}}(C)$$

thus justifying our regression-based approach.

### Computing the effect of α on sampling strategy efficiency

To compute the number of recovered clusters in breast cancer and cardiac tissue as a function of both *r* (number of regions) and *w* (width of FoV), we substituted equation (2) into equation (1):

$$N\left( {r,w} \right) = N_{\mathrm{O}}\left( {1 - \exp \left( {\frac{{rw^{\upalpha}}}{C}} \right)} \right)$$

To compare both samples, we dropped the *N*_O_ term. We then selected three total area values (1.6 mm^2^, 0.8 mm^2^ and 0.32 mm^2^) and computed *N*(*r*,*w*) for different ratios of *r* and *w* with a constant *r*×*w*^2^ (total area) value.

### Reporting summary

Further information on research design is available in the Nature Portfolio Reporting Summary linked to this article.

## Online content

Any methods, additional references, Nature Portfolio reporting summaries, source data, extended data, supplementary information, acknowledgements, peer review information; details of author contributions and competing interests; and statements of data and code availability are available at 10.1038/s41592-022-01692-z.

## Supplementary information

Supplementary Information Practical guide to optimize multiplexed imaging experiment.

Reporting Summary

Supplementary Table 1 List of spatial transcriptomic datasets used in the manuscript.

Supplementary Table 2 IMC panels for lymph node imaging.

Supplementary Table 3 Computed values of alpha for various human healthy and tumor tissues.

## Data Availability

Raw and processed human lymph node IMC datasets generated for this article are freely available on Mendeley (https://data.mendeley.com/datasets/ncfgz5xxyb/1). Spatial transcriptomic data were either downloaded from the 10x website (support.10xgenomics.com/spatial-gene-expression/datasets/) or from the Gene Expression Omnibus (GEO) repository (see Supplementary Table 1). CosMX lung cancer data were downloaded from the nanoString website (https://nanostring.com/products/cosmx-spatial-molecular-imager/ffpe-dataset/). Source data has been provided for Figs. 1 and 2 and Extended Data Figs. 1 and 2.

## Source data

Source Data Statistical source data for Figs. 1 and 2 and Extended Data Figs. 1 and 2.

## Code availability

All scripts and code used in this paper are available on GitHub at https://github.com/BodenmillerGroup/MI_Sampling_study.
