# Supplementary material for: Optimizing multiplexed imaging experimental design through tissue spatial segregation estimation
Source: Nat Methods. 2022 Dec 30;20(3):418–23. doi: 10.1038/s41592-022-01692-z (PMC9998266; doi:10.1038/s41592-022-01692-z)
Supplement: Supplementary file 1 — Practical guide to optimize multiplexed imaging experiment. [file 41592_2022_1692_MOESM1_ESM.pdf]

nature methods

Article https://doi.org/10.1038/s41592-022-01692-z

Optimizing multiplexed imaging experimental design through tissue spatial segregation estimation

In the format provided by the authors and unedited

Practical consideration for multiplexed imaging experimental design

This document contains practical guidelines for the experimental design of multiplexed imaging experiments, for instance Imaging Mass Cytometry experiments. We will consider that the experiment involves a homogenous group of samples, all derived from the same type of tissue/organ. In practice, the imaging time required to image the whole area of all samples is too long, therefore one should determine the minimal area to image per sample such that the global composition of each sample can be accurately described. Here we will describe three practical cases where our model can be used together with prior experimental data to optimize experimental design. All scripts and functions used here are available on a GitHub repository (https://github.com/PierreBSC/MI_Sampling_study).

A) Derive sampling parameters from a large multiplexed panorama image

The experimenter can image a large region, typically several mm^2, of a representative sample with the same technology as the one that will be used for all samples in the experiment (and in the case of a targeted approach, the same marker panel). The resulting data can then be analyzed using our method (Equations (1) and (2) in Bost et al; implemented in our script by the R function Perform_sampling_analysis()). It will determine the $\boldsymbol{\tau}$ value for various FoV sizes and the relation between the two, i.e the $\boldsymbol{\alpha}$ parameter. Once the FoV size has been selected, we recommend to image $2\boldsymbol{\tau}$ FoVs of the selected FoV size in order to reach a reasonable saturation (>86% of the cell phenotype recovered on average). While this is the most quantitative and rigorous approach, it requires a preliminary experiment, thus representing additional reagent cost and imaging time.

B) Infer sampling parameters from previous multiplex imaging data

In some cases, the experimenter will have access to previously generated multiplexed imaging data from the same tissue/organ. The technology used to generate this dataset should be the same as the one planned to be used to generate new data, including a similar marker panel. In most cases those data will consist of a set of small FoVs, that can be used to infer the $\boldsymbol{\alpha}$ parameter value through a shrinkage analysis, as described in the manuscript (Figure S2g). This is done using the Global_alpha_estimation() function and will provide a rough estimate of $\boldsymbol{\alpha}$. While this approach does not provide a precise description of the optimal sampling strategy, it can still identify tissues

with a low α value (i.e., with low spatial segregation) where imaging numerous small FoV should be highly favored compared to imaging a small number of large FoVs. In practice, we recommend to image FoVs with a width lower than 200µm when the α is equal to or below one, such as in the highly structured breast cancer samples.

C) Infer sampling parameters from publicly available spatial transcriptomic (ST) data

If ST data (e.g., Visium data) from the tissue/organ of interest are publicly available, the datasets can be first processed using the R script Visium_data_processing.R and then analyzed using the Perform_sampling_analysis() function in order to estimate the value of the α parameter. The computed τ value cannot be directly used, due to the difference of technology, as illustrated in the manuscript (Figure 2c). As for strategy B above, this approach should be used to identify tissues with a low α value (i.e., with low spatial segregation) where imaging numerous small FoV should be highly favored compared to imaging a small number of large FoVs.

Note that our paper (Bost et al) contains a list of computed α values (i.e., degree of cell phenotype spatial segregation within the tissue) for various healthy and tumor tissues (Supplementary Table 3) using publicly available ST Visium datasets.

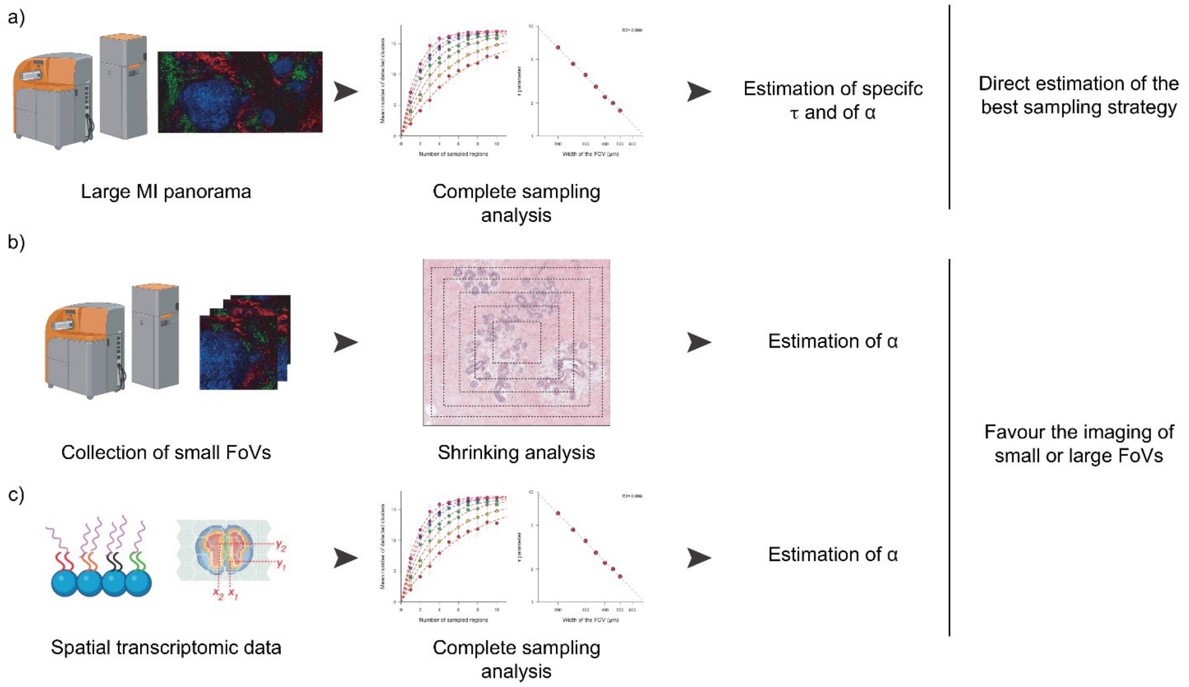

Reference

Optimizing multiplexed imaging experimental design through tissue spatial segregation estimation.

Bost P., Schulz D., Engler S., Wasserfall C. and Bodenmiller B. (2022). Nature Methods.
