# Supplementary material for: Optimizing multiplexed imaging experimental design through tissue spatial segregation estimation
Source: Nat Methods. 2022 Dec 30;20(3):418–23. doi: 10.1038/s41592-022-01692-z (PMC9998266; doi:10.1038/s41592-022-01692-z)
Supplement: Supplementary file 2 — Reporting Summary [file 41592_2022_1692_MOESM2_ESM.pdf]

nature portfolio

Corresponding author(s): Bernd Bodenmiller

Last updated by author(s): Aug 17, 2022

Reporting Summary

Nature Portfolio wishes to improve the reproducibility of the work that we publish. This form provides structure for consistency and transparency in reporting. For further information on Nature Portfolio policies, see our Editorial Policies and the Editorial Policy Checklist.

Statistics

For all statistical analyses, confirm that the following items are present in the figure legend, table legend, main text, or Methods section.

n/a	Confirmed	
☐	☒	The exact sample size (*n*) for each experimental group/condition, given as a discrete number and unit of measurement
☒	☐	A statement on whether measurements were taken from distinct samples or whether the same sample was measured repeatedly
☐	☒	The statistical test(s) used AND whether they are one- or two-sided<br>*Only common tests should be described solely by name; describe more complex techniques in the Methods section.*
☒	☐	A description of all covariates tested
☐	☒	A description of any assumptions or corrections, such as tests of normality and adjustment for multiple comparisons
☐	☒	A full description of the statistical parameters including central tendency (e.g. means) or other basic estimates (e.g. regression coefficient) AND variation (e.g. standard deviation) or associated estimates of uncertainty (e.g. confidence intervals)
☐	☒	For null hypothesis testing, the test statistic (e.g. *F*, *t*, *r*) with confidence intervals, effect sizes, degrees of freedom and *P* value noted<br>*Give P values as exact values whenever suitable.*
☒	☐	For Bayesian analysis, information on the choice of priors and Markov chain Monte Carlo settings
☒	☐	For hierarchical and complex designs, identification of the appropriate level for tests and full reporting of outcomes
☐	☒	Estimates of effect sizes (e.g. Cohen's *d*, Pearson's *r*), indicating how they were calculated

Our web collection on statistics for biologists contains articles on many of the points above.

Software and code

Policy information about availability of computer code

Data collection	CYTOF 7.0 software was used for IMC data acquisition.
Data analysis	IMC images were processed using Steinbock version 0.7 (bodenmillergroup.github.io/steinbock). Resulting data were analyzed using R version 4.0.3 and in-house scripts. Visium data were processed using the pagoda2 1.0.0 R package. All scripts developed for this paper are available on a GitHub repository (https://github.com/PierreBSC/MI_Sampling_study).

For manuscripts utilizing custom algorithms or software that are central to the research but not yet described in published literature, software must be made available to editors and reviewers. We strongly encourage code deposition in a community repository (e.g. GitHub). See the Nature Portfolio guidelines for submitting code & software for further information.

Data

Policy information about availability of data

All manuscripts must include a data availability statement. This statement should provide the following information, where applicable:

- Accession codes, unique identifiers, or web links for publicly available datasets
- A description of any restrictions on data availability
- For clinical datasets or third party data, please ensure that the statement adheres to our policy

The raw and processed human lymph node IMC datasets are available on a Mendeley repository (https://data.mendeley.com/datasets/ncfgz5xxyb/1).

Human research participants

Policy information about studies involving human research participants and Sex and Gender in Research.

Reporting on sex and gender	*Use the terms sex (biological attribute) and gender (shaped by social and cultural circumstances) carefully in order to avoid confusing both terms. Indicate if findings apply to only one sex or gender; describe whether sex and gender were considered in study design whether sex and/or gender was determined based on self-reporting or assigned and methods used. Provide in the source data disaggregated sex and gender data where this information has been collected, and consent has been obtained for sharing of individual-level data; provide overall numbers in this Reporting Summary. Please state if this information has not been collected. Report sex- and gender-based analyses where performed, justify reasons for lack of sex- and gender-based analysis.*
Population characteristics	*Describe the covariate-relevant population characteristics of the human research participants (e.g. age, genotypic information, past and current diagnosis and treatment categories). If you filled out the behavioural & social sciences study design questions and have nothing to add here, write "See above."*
Recruitment	*Describe how participants were recruited. Outline any potential self-selection bias or other biases that may be present and how these are likely to impact results.*
Ethics oversight	*Identify the organization(s) that approved the study protocol.*

Note that full information on the approval of the study protocol must also be provided in the manuscript.

Field-specific reporting

Please select the one below that is the best fit for your research. If you are not sure, read the appropriate sections before making your selection.

☒ Life sciences ☐ Behavioural & social sciences ☐ Ecological, evolutionary & environmental sciences

For a reference copy of the document with all sections, see nature.com/documents/nr-reporting-summary-flat.pdf

Life sciences study design

All studies must disclose on these points even when the disclosure is negative.

Sample size	As no comparison between two groups of sample was performed, no sample size was determined.
Data exclusions	No data exclusion was performed.
Replication	We performed a large-scale imaging of two human lymph node coming from two-different healthy donors in order to validate our approach.
Randomization	There was no analysis comparing different groups, therefore no randomization was done.
Blinding	There was no analysis comparing different groups, therefore no blinding was necessary.

Reporting for specific materials, systems and methods

We require information from authors about some types of materials, experimental systems and methods used in many studies. Here, indicate whether each material, system or method listed is relevant to your study. If you are not sure if a list item applies to your research, read the appropriate section before selecting a response.

Materials & experimental systems

n/a	Involved in the study	
☐	☒	Antibodies
☒	☐	Eukaryotic cell lines
☒	☐	Palaeontology and archaeology
☒	☐	Animals and other organisms
☒	☐	Clinical data
☒	☐	Dual use research of concern

Methods

n/a	Involved in the study	
☒	☐	ChIP-seq
☒	☐	Flow cytometry
☒	☐	MRI-based neuroimaging

Antibodies

Antibodies used

MPO : Rabbit polyclonal (ab9535) from Abcam
CD8a : C8/144B clone from Abcam (ab17147) and clone RPA-T8 from Biolegend (301002)
SMA : 1A4 clone from Abcam (ab7817)
CCR7 : EPR23192-57 from Abcam (ab253187)
HLA-DR : TAL 1B5 clone from Abcam (ab20181)
ICOS : D1K2T clone from Cell Signaling (#89601)
XBP1 : polyclonal from ThermoFisher (# PA5-27650)
Histone H3K9Ac : C5B11 clone from Cell Signaling (#9649)
HIF-1a : EP1215Y clone from Abcam (ab51608)
CD20 : L26 clone from Abcam (ab9475)
CD163 : EDHu-1 clone from Thermofisher (MA1-82342)
CD56 : E7X9M clone from Cell Signaling (#99746)
Granzyme B : D6E9W clone from Cell Signaling (# 46890)
Human IgM : MHM-88 clone from Biolegend (314502)
LEF1 : EPR2029Y clone from Abcam (ab137872)
CD69 : EPR21814 clone from Abcam (ab233396)
MMP9 : D6O3H clone from Cell Signaling (#13667)
CD40L : EP462E clone from Abcam (ab210743)
PD-L1 : 73-10 clone from Abcam (ab226766)
CD45RO : UCHL1 clone from Biolegend (304202)
FOXP3 : 236A/E7 clone from Thermofisher (# 14-4777-82)
CXCL13 : Goat polyclonal from R&D Systems (AF801)
CD9 : D3H4P clone from Cell Signaling (#13403)
CD3 : Rabbit polyclonal from Agilent (A0452)
GITR : D5V7P clone from Cell Signaling (#10419)
CD303 : Goat polyclonal from R&D Systems (AF1376)
CD209 : C209/1781 clone from Thermofisher (#30835-MSM1-P1ABX)
AICDA : EPR23436 clone from Abcam (ab269457)
MX1 : D3W7I clone from Cell Signaling (#37849)
CCL21 : Goat polyclonal from R&D Systems (AF366)
CD31 : EPR3094 clone from Abcam (ab76533)
Cleaved Caspase3 : C92-605 clone from BD Biosciences (559565)
CCL19 : 54909 clone from Thermofisher (# MA5-23833)
Caveolin-1 : D46G3 clone from Cell Signaling (#3267)
Vimentin : EPR3776 clone from Abcam (ab193555)
Ki67 : B56 clone from BD (556003)
IDO : SP260 clone from Abcam (ab228468)
TCF1/TCF7 : C63D9 clone from Cell Signaling (#2203)
BTLA : EPR22224-271 clone from Abcam (ab230976)

Validation

MPO : validated on human B-cell lymphoma (IHC).
CD8a : validated on human tonsil (IHC)
SMA : validated on human breast ductal carcinoma tissue (IHC).
CCR7 : validated on human tonsil (IHC).
HLA-DR : validated on human tonsil (IHC) and human skin (IHC).
ICOS : validated on human tonsil (IHC) and human lymphoma (IHC).
XBP1 : validated on human breast carcinoma (IHC).
Histone H3K9Ac : validated on human gastic carcinoma (IHC) with or without competitor peptide (K9 acetyl-peptide).
HIF-1a : validated on hypoxic and normoxic region of human colorectal cancer (IHC).
CD20 : validated on human tonsil (IHC).
CD163 : no validation provided by the manufacturer but obtained signal corresponds to the expected shape of macrophage in a human lymph node.
CD56 : validated on peripheral nerve from a human prostate adenocarcinoma (IHC).
Granzyme B : validated in human colon adenocarcinoma (IHC).
IgM : validated on human PBMC with a CD19 co-staining (flow-cytometry)
LEF1 : validated on human tonsil (IHC) and Jurkat cells (IF)
CD69 : validated on human tonsil (IHC) and human cervix cancer (IHC)
MMP9 : validated on stimulated and unstimulated U-2 OS cells (IHC) and human breast carcinoma (IHC).
CD40L : validated on human lymphoid tissue lysate (WB)
PD-L1 : validated on human tonsil (IHC).
CD45RO : validated on human PBMCs (flow-cytometry).
FOXP3 : validated on human PBMCs with a CD25 co-staining (flow-cytometry).
CXCL13 : validated on human lymphoma (IHC).
CD9 : validated on human breast carcinoma (IHC).
CD3 : validated on Jurkat cells lysate (WB).
GITR : validated on human colon carcinoma (IHC).
CD303 : validated on human tonsil (IHC).
CD209 : validated on human small intestine (IHC).
AICDA : validated on human B-cell and Hodgkin's lymphoma (IHC).

MX1 : validated on human ductal carcinoma (IHC).
CCL21 : validated on human PBMC (IF).
CD31 : validated on human kidney (IHC).
Caspase-3 : validated on treated and untreated Jurkat cells (flow-cytometry).
CCL19 : validated on human tonsil (IHC).
Caveolin-1 : validated on human colon carcinoma (IHC).
Vimentin : validated on human cervical carcinoma (IHC).
Ki67 : validated on U-2 OS, A549 and HeLa cells (IF).
IDO1 : validated on human tonsil (IHC).
TCF1/TCF7 : validated on Jurkat cells (flow-cytometry).
BTLA : validated on human tonsil (IHC).
